# Effect of laboratory manual layout: does experiential learning benefit from authentic context?

**DOI:** 10.1099/acmi.0.000955.v4

**Published:** 2025-06-17

**Authors:** Victoria Alice Kate Easton

**Affiliations:** 1 School of Molecular and Cellular Biology, University of Leeds, Leeds, UK

**Keywords:** authentic context, authentic learning, experiential learning (EL), practical teaching

## Abstract

Experiential learning is the pedagogic foundation of practical laboratory education. This process of learning through experience enables students to develop a deeper understanding of the theoretical material as well as valuable real-world skills. However, there is often a disconnect between the authentic, real-world context of performing laboratory skills and the method of instruction within higher education. This study developed two student laboratory manuals; one which followed a traditionally linear ‘week by week’ format, and another which took inspiration from a publication format and listed the protocols in a distinct ‘methods’ section. The effect the change of layout had on student learning was assessed through analysis of student summative assessment and interaction with the online learning environment. Additionally, the effect on student confidence and perceived technical skills development was assessed through a student survey. The differences in layout resulted in no significant differences in student assessment performance but did result in higher levels of engagement with the online learning environment. The student survey reported an increase in technical confidence (21%) and skill (31%) with the authentic ‘methods’ section layout changes compared to the traditional format. The increase in student engagement, confidence and perceived skill shows that experiential learning benefits from placing the information in an authentic context.

## Data Summary

The authors confirm that all supporting data, code and protocols have been provided within the article or through supplementary data files.

## Introduction

Practical laboratory teaching provides a holistic approach to learning, combining theoretical knowledge with experiences. It has been shown to enhance understanding [1,2], and this aspect of contextual learning can allow for a more direct progression to post-university employment [3]. Practical teaching can significantly enrich the educational experience and better prepare students for future academic and professional endeavours.

Experiential learning (EL) in the context of practical laboratory teaching is a hands-on approach to education that emphasizes learning through experience and reflection.

The history of EL [4,5] can be traced back to the philosopher and educator John Dewey [6] and can take various forms such as encouraging active participation with students not just as passive observers but actively engaging; problem-solving where students are presented with a problem to solve or a hypothesis to test; and skills development where students develop not just subject-specific technical skills, but also transferable skills like critical thinking, collaboration and communication.

The goal is to bridge the gap between theory and practice, allowing students to understand the real-world applications of what they learn. By doing so, students can develop a deeper understanding of the subject matter, experience better learning outcomes [7] and are better prepared for professional practice after graduation [8]. Experiential learning in laboratories also fosters independence, confidence and technical competencies, as students learn to trust their abilities to conduct experiments and interpret results.

In essence, EL aims to be an active, student-centred environment where learning is a dynamic and interactive process. Unsurprisingly, EL is an ongoing consideration when trying to improve learning outcomes in higher education [9,11].

Authenticity within a higher education context often refers to the genuine and transparent engagement of students and educators in the learning process. Authenticity in education is most commonly linked to assessment, where it can improve the learning experience of students and their engagement, as well as develop professional or work-ready skills [12]. It can relate to authentic teachers [13], which, according to Palmer, relates to teachers finding their ‘integrity’ [14]. However, less well developed is the idea of an authentic teaching context – a concept involving authentic activities and environments [15,16].

Authentic activities and environments are umbrella terms, with the authentic context being a method with which to achieve them. Authentic contexts will present a realistic structure and preserve the complexity of real-life processes [17]. Students should be able to access the information and resources in an authentic context rather than dis-embedding course materials from the genuine practice of professionals [18].

With regard to practical laboratory education, authentic contexts include practical laboratory manuals. Traditionally, this information is presented in a linear ‘week by week’ format, which does not mimic how a graduate scientist would interact with methods in a professional setting. To explore best practice, two lab manuals were developed. One follows a linear description of practicals performed over the course of the year. The other displays the information along the lines of a scientific paper, with the descriptive explanatory text referring to a subsequent methods section.

The project aims to evaluate how the presentation of information in practical laboratory manuals impacts their effectiveness. Does a more authentic context build student confidence in their technical abilities, increase engagement or result in better student outcomes?

## Methods

The participants for this study were 96 first-year students of a 3-year undergraduate programme taking a compulsory skills module within the School of Molecular and Cellular Biology at the University of Leeds. These students take two concurrent strands of practical work (molecular biology and microbiology). The practical laboratory manuals for these two strands were designed differently. The molecular biology strand [no intervention (NI)] retained the traditional week-by-week format for their 3 h practical each week (see Fig. S1, available in the online Supplementary Material), while the microbiology strand contained a separate ‘methods’ section to simulate a scientific paper (intervention (I)] for their weekly 3 h practical (see Fig. S2). The information each manual contained was not changed, only the layout. All students experienced both styles of manual for the full academic year. Table 1 shows the breakdown of participants for each metric analysed.

**Table 1.** Student participation within the project, including a year of study participation rate

Analysis	Year	Student year of study	Participant/module maximum*	Participation rate
**Student survey (NI**)	2023/24	First year	49/96	51%
**Student survey (I**)	2023/24	First year	51/96	53%
**Student engagement**	2023/24	First year	96/96	n/a
	2022/23†	First year	104/104	n/a
**Student outcomes (NI**)	2023/24	First year	91/96	95%
**Student outcomes (I**)	2023/24	First year	91/96	95%

*The number of students enrolled in the module.

†No changes to the material available online were made.

### Student survey

At the end of the academic year, students were offered the opportunity to reflect on their experiences with the two different styles of manuals. Surveys relating to the NI and I were offered, which comprised two Likert scales containing five points (strongly disagree, disagree, neither agree nor disagree, agree and strongly agree). The questions posed for each survey were as follows:

1. This style of manual increased my confidence.
2. This style of manual increased my technical skill.

To avoid bias when identifying the NI and I manuals, they were referred to as ‘week-by-week’ manuals and ‘methods section manuals’, retrospectively.

### Student engagement with online resources

Attendance at laboratory practical sessions is compulsory and so a poor representation of engagement. Instead, the total number of hours spent on the online learning environment for the module was used as a representation of student engagement. The total number of hours each student spent during the intervention year was compared to the prior year, and an unpaired t-test with Welch’s correction was performed. This was decided as part of the experimental planning phase, as no assumption could be made that the two populations would have the same standard deviation [19,20]. Significance was set at *P*<0.05. It should be noted that the module manager did change between these years, but the academic leading the practical sessions remained the same. No other notable changes were made to the student experience.

### Student outcomes

The module used for this project contains two concurrent strands of learning, focusing primarily on practical molecular biology (NI) and practical microbiology (I). This module also contains tutorials and seminars relating to associated scientific theory and academic skills development, which were not investigated in this project. A practical skills assessment is used to assess each strand and is undertaken by all students. The marks achieved by the students in each of these assessments were compared, and an unpaired t-test with Welch’s correction was performed. This was decided as part of the experimental planning phase, as no assumption could be made that the two populations would have the same standard deviation [19,20]. Significance was set at *P*<0.05. The practical skills assessment is not the sole assessment method: report writing, oral presentations, multiple response questions and essays were also credit-bearing assessments. The specific assessments differ by degree programme, except the skills assessment, which was taken by all students and, as such, was used in this analysis.

## Results

In order to explore whether experiential learning benefits from authentic contexts, two practical laboratory manuals were generated. All participants experienced both styles of manual, and no content changes were made to ensure that the students were not negatively impacted by this research.

### Student survey

The student’s lived experience is always important to consider, even just as a consultatory voice [21]. Students were offered the chance to reflect on their learning experiences with the NI and I manuals. In order to maximize response rates, ultra-short questionnaires were developed [22], which contained just two Likert scales. The ultra-short questionnaires received 51% (NI) and 53% (I) response rates (Table 1).

For ease of interpretation, statements of general agreement (agree and strongly agree) and statements of general disagreement (disagree and strongly disagree) were combined (Table 2). The I manual with the separate methods section resulted in student responses of 90% combined agreement, with 57% of students agreeing and 33% strongly agreeing with the statement – ‘This style of manual increased my confidence’. The NI manual with the traditional linear format resulted in student responses of 69% combined agreement, with 51% of students agreeing and 18% strongly agreeing with the same statement. The I manual, therefore, resulted in a 21% increase in student confidence with laboratory practicals, with the largest difference seen in the strongly agreeing category.

**Table 2.** Survey data of student perceptions of NI and I manuals on confidence and technical skill

	Aspect investigated	Strongly disagree (%)	Disagree (%)	Combined disagreement (%)	Neither (%)	Agree (%)	Strongly agree (%)	Combined agreement (%)	I-NI*
**No intervention**	Confidence	0 (0%)	11 (22%)	22%	4 (8%)	25 (51%)	8 (18%)	69%	21%
**Intervention**		0 (0%)	2 (4%)	4%	3 (6%)	29 (57%)	17 (33%)	90%	
**No intervention**	Technical skill	3 (6%)	5 (10%)	16%	9 (18%)	24 (49%)	8 (16%)	65%	31%
**Intervention**		0 (0%)	1 (2%)	2%	1 (2%)	23 (63%)	17 (33%)	96%	

*The percentage difference between the NI and the I combined agreement.

The I manual resulted in student responses of 96% combined agreement, with 63% of students agreeing and 33% strongly agreeing with the statement – ‘This style of manual increased my technical skill’. The NI manual resulted in student responses of 65% combined agreement, with 49% of students agreeing and 16% strongly agreeing with the same statement. The I manual, therefore, resulted in a 31% increase in student perceived technical skill, with the largest difference seen in the strongly agreeing category.

### Student engagement with the online learning environment

**Fig. 1.**
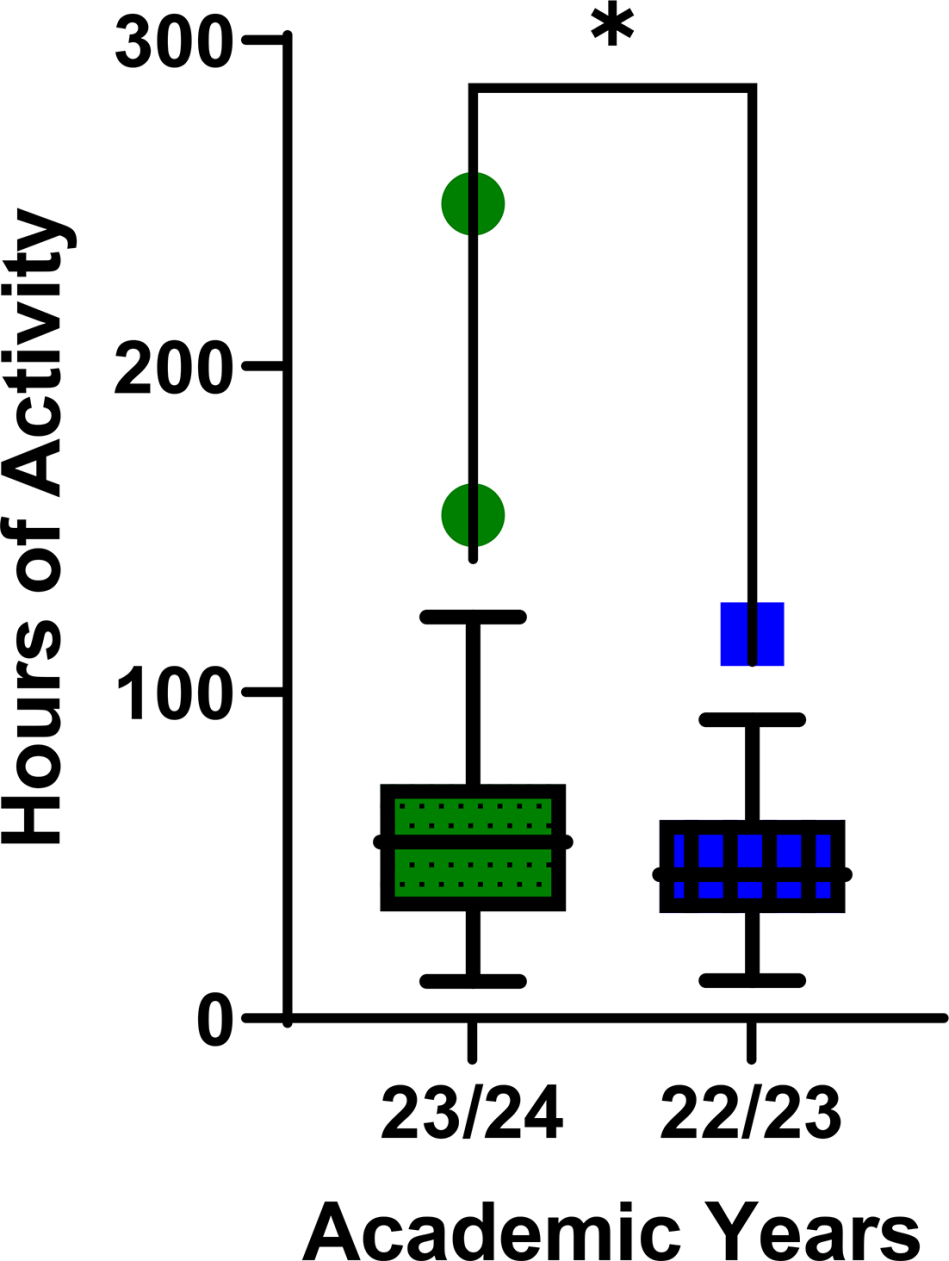
Student engagement with the online learning environment. A box and whisker plot showing the distribution of student hours (**h**) of activity on the module for the NI/I year (23/24) and the NI only year (22/23). Data were compared using an unpaired two-tailed t-test with Welch’s correction (*P-value<0.05). Outliers for the 23/24 year and the 22/23 year (green circles and blue squares, respectively) were omitted from the analysis. A table containing the values represented in the graph above can be found as Table. S1, A.

Student engagement is a nuanced metric to analyse, but one that the author felt was important to investigate, given that engagement has been seen to increase with both EL and authentic learning. Attendance as a proxy for engagement, due to measuring the quantity of learners’ active participation [23], is not possible here, as practical classes are mandatory. The amount of time spent on the online learning environment for the module was used as a representation of student engagement (Fig. 1). Data were derived from the learning analytics generated by the online learning platform. The module in which this project is based contains a single online learning area for both the molecular biology (NI) and microbiology (I) strands of practical work; therefore, it was not possible to differentiate engagement with the NI and I content. Instead, the academic year in which the project was undertaken (both NI and I) was compared to the previous year (NI only).

The cohorts for these 2 years (22/23 and 23/24) were of similar size (96 and 104, respectively) (Fig. 1); a table of the data discussed below can be found in Table S1, A. The lowest total number of hours of activity on the module and the lower quartile are similar for both the NI/I year (23/24) and the NI only year (22/23). The mean number of active hours is, however, significantly different (*P*-value=0.0192), with the NI/I year (23/24) receiving 56.8 hours/year and the NI only year (22/23) receiving 47.5 hours/year. The median and upper quartile hours of activity are also higher for the NI/I year (23/24). This suggests that the students from the NI/I year (23/24) are more engaged than the previous year.

There was no difference in content between these years, simply a change in layout towards placing that information in an authentic context.

### Student outcomes

Assessment outcomes were monitored as effective experiential learning has been seen to improve student performance [24], although there is controversy with this statement, as some published work disputes this link [25].

**Fig. 2.**
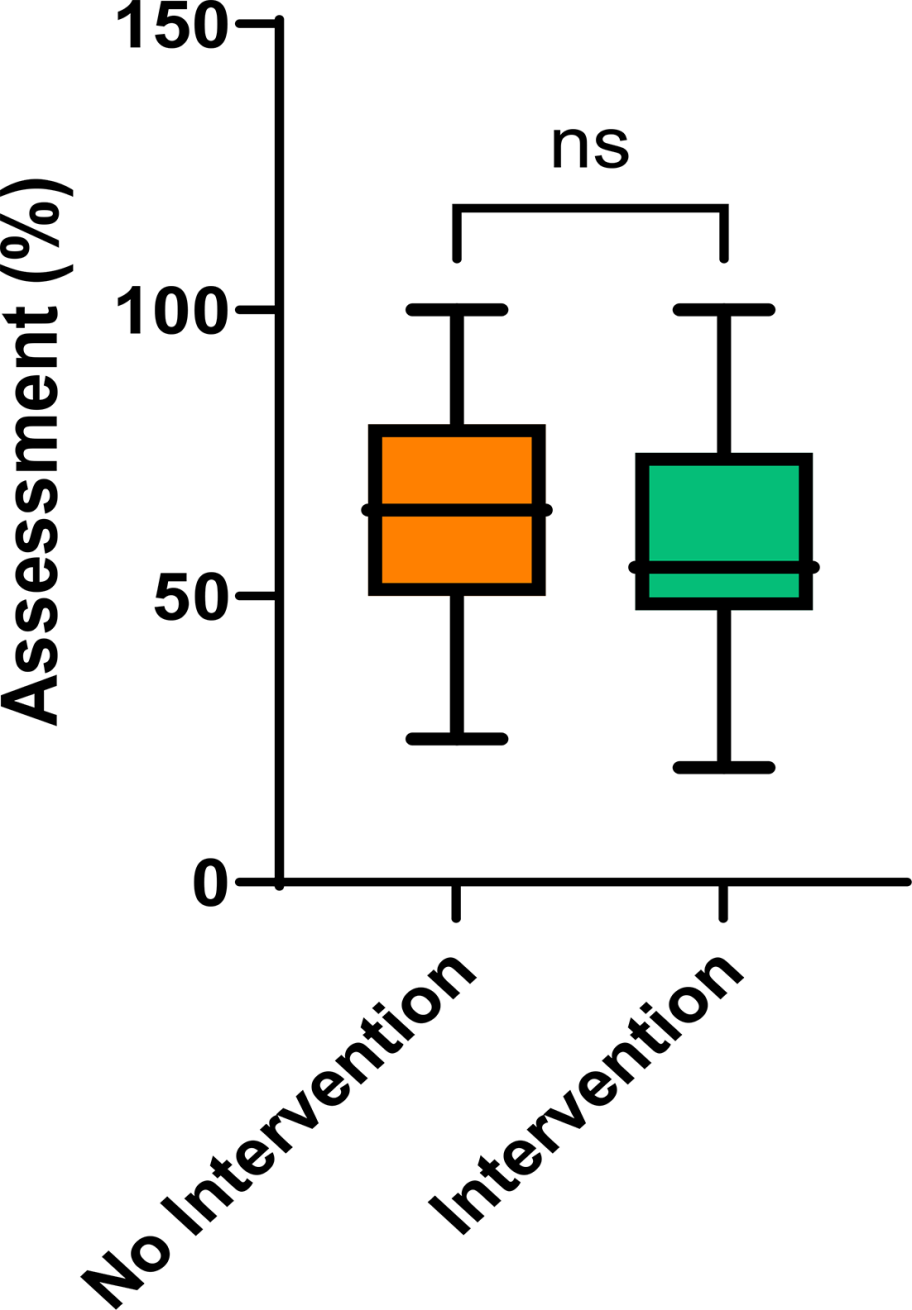
Student outcomes of practical skills assessment. A box and whisker plot showing the distribution of student results for the NI and I skills assessments. Data were compared using an unpaired two-tailed t-test with Welch’s correction (*P-value<0.05). A table containing the values represented in the graph above can be found as Table S1, B.

The results of the skills assessment for the molecular biology (NI) and microbiology (I) stands were compared (Fig. 2). Student results range from 20% to 100% (I) and 25% to 100% (NI). The mean student result for the NI assessment was higher than the I assessment (64.8% and 59.7%, respectively), but this difference was not statistically significant (*P-*value=0.0509). This suggests that this project’s interpretation of authentic context did not have an effect on student performance.

## Discussion

This study aimed to explore whether experiential learning benefits from authentic contexts. The hypothesis is that by providing an authentic context that reflects the way the knowledge will be used in real life, it will result in a synergistic effect when coupled with experiential learning.

To achieve this, two practical laboratory manuals were created: one following a traditional linear format (no intervention group, NI) and the other mimicking a scientific paper layout (intervention group, I).

Two students facing ultra-short questionnaires were developed to maximize response rates. These questionnaires contained only two Likert scales. Response rates for the NI (51%) and I (53%) surveys (Table 1) were high compared to the usual rate within the School of Cellular and Molecular Biology (personal communications). They are also higher than the average online survey response rate of 44.1% [26], suggesting that the selection of ultra-short questionnaires was beneficial in maximizing response rates.

Low response rates are more susceptible to bias, and the results can be unrepresentative of the group [27]. The data generated here, however, is likely to be representative given the work of Nulty [28]. The course, which was used in this study, had 96 students (23/24 academic year). The required response rate for ‘liberal conditions’ of adequate response rate to support adequate evidence of consensus would be 21%. This study exceeds this estimate considerably in both the NI and I survey response rates (Table 1).

However, the decision to use ultra-short questionnaires is not without limitations. It has led to a limited dataset from which the nuance behind student perceptions of ‘confidence’ and ‘technical skill’ cannot be investigated. A follow-on study involving listening rooms and round table analysis is currently planned as they present a safe space to explore lived experiences and place the student voice and stakeholder experience at the forefront of analysis [29].

The combined statements of agreement of the I manual resulted in 90% combined agreement (responses of agreed and strongly agreed) regarding increased confidence (Table 1). In contrast, the NI manual achieved 69% combined agreement. Changing the manual layout for this experiential learning activity to present the information in a more authentic context led to a 21% increase in student confidence, with the largest difference observed in the strongly agreeing category, emphasizing the strength of student belief that the manuals contributed to their confidence.

Student confidence is a teaching metric that should not be ignored as it plays a large role in better student outcomes [30,31]. Equally, student performance can be improved by reducing stress and anxiety within the practical laboratory [32]. The work here demonstrates that the changes in the layout of the laboratory manual to reflect an authentic context have increased student confidence.

Similarly, the I manual had a positive impact on perceived technical skill. It resulted in 96% combined agreement that it enhanced their technical abilities, while the NI manual achieved 65% combined agreement. Overall, the I manual led to a 31% increase in perceived technical skill compared to the NI manual, with the strongest difference seen in the strongly agreeing category. These findings suggest that the intervention group (I) with the separate methods section positively influenced both student confidence and technical skill. The authentic context provided by the scientific paper layout appears to enhance the experiential learning experience.

It should be noted that the combined agreement for the NI manual was high. The data do not suggest that the traditional format is detrimental to student confidence or perceived skill development. It does, however, clearly demonstrate that the students favoured the authentic context the experiential learning was framed within. Upon reflection, this could be due to the independence gained from the process of identifying the correct method within the authentic context [33], as this has been suggested to increase confidence, or that separating the method from the theory benefits experiential learning, as students can focus on the physical actions they are undertaking.

In this study, attendance could not serve as a proxy for engagement due to mandatory practical classes. Instead, the amount of time students spent on the online learning environment for the module was used as an indicator of engagement. The engagement data could be analysed and compared to the previous year (22/23) when a traditional, linear format was used for both molecular biology and microbiology strands. It is important to note that no changes were made to the learning materials available on the online learning environment or the members of staff teaching on the module between the 2 years. Despite this, the findings support the hypothesis that placing experiential learning information in an authentic experience can promote student engagement.

The cohorts for the 2 years (22/23 and 23/24) were similar in size (96 and 104 students, respectively). The lowest total number of engagement hours and the lower quartile (Q1) of engagement were comparable between the NI/I year (23/24) and the NI-only year (22/23). However, the mean number of active hours significantly differed (*P-*value=0.0192) between the NI/I year (23/24) and the NI-only year (22/23).

This suggests that students in the NI/I year (23/24) were more engaged with the online learning environment than in the previous year. The NI/I year (23/24) also exhibited higher median and upper quartile hours of activity. The shift in layout appears to have contributed to increased online engagement, even without altering the content available either online or within the laboratory manuals themselves.

Interestingly, the least engaged students demonstrated no increase in engagement with the intervention. It is the students who are already engaged in the experiential learning process (mean and Q3), who seem to benefit from the inclusion of authentic context the most. To develop upon this work, the question of how to increase the engagement of the least engaged students must be addressed.

Only a single year’s worth of comparative data was included in this study, as there were concerns regarding the relevance of the 21/22 or 20/21 academic years due to the shift in online learning during the SARS-CoV-2COVID-19 pandemic. Future work in this area could expand the analysis over multiple years to increase statistical confidence. Ultimately, student engagement data gathered from an online learning environment may be flawed as it only shows time logged with an open page – not actual learning. Additionally, personal communication with students has reported that some students access the information together as part of study groups. While the data generated from the online learning environment does have limitations, it is believed that it can be used as a proxy for engagement in lieu of any additional metric available. The use of learning analytics generated by online learning environments is an emerging research area [34], which can generate meaningful information on student behaviours in online learning environments, which, in turn, reflects the influence of learning design on the teaching and learning process. An aspect of importance for this study. However, planned future work involving listening rooms could be used to highlight the students’ perceptions of engagement and see if they align with the data produced by the online learning analytics.

In comparing the results of the skills assessments for molecular biology (NI) and practical microbiology (I), it was observed that student performance varied widely, with scores ranging from 20% to 100% for practical microbiology and 25% to 100% for molecular biology. The mean scores for the NI assessment were slightly higher than those for the I assessment (64.8% vs. 59.7%). However, this difference was not statistically significant (*P-*value=0.0509), suggesting that the inclusion of experiential learning information in an authentic context did not significantly affect student performance.

Authentic assessment is valuable for providing practical skills and real-world applications [12,35, 36]. However, in this small study, the impact of authentic context on overall student performance in experiential learning assessment may not be as pronounced as that seen in other publications. Upon reflection, this could be due to various factors, such as the nature of the assessments and the students’ familiarity with the practical tasks or the specific content of the modules. Further research is needed to explore these variables and to fully explore the role that placing experiential learning in authentic contexts plays. Ultimately, the data generated here adds to the controversy [24,25] over whether effective experiential learning can result in increased student assessment performance. Expanding the study to cover different subjects and assessment styles needs to be investigated to fully clarify the interplay between authentic context, experiential learning and student performance in assessment.

Within the scope of this project, the changes to manual layout may not have resulted in increased student assessment performance, but the strong preference displayed in the student surveys and the increase in engagement demonstrate that experiential learning does benefit from authentic contexts.

## Supplementary material

10.1099/acmi.0.000955.v4 Uncited Supplementary Material 1.

## Abbreviations

EL: experiential learning
I: intervention
NI: no intervention

## References

[1] Garnett PJ, Tobin K (1989). Teaching for understanding: exemplary practice in high school chemistry. *J Res Sci Teach*.

[2] Kolb AY, Kolb DA (2017). Learning styles and learning spaces: enhancing experiential learning in higher education. https://journals.aom.org/doi/abs/10.5465/AMLE.2005.17268566.

[3] Osika A, MacMahon S, Lodge J, Carroll A (2022). Contextual learning: benefits and examples. https://www.timeshighereducation.com/campus/contextual-learning-linking-learning-real-world.

[4] Kolb DA (1984). Experiential learning: experience as the source of learning and development. https://books.google.com/books/about/Experiential_Learning.html?id=o6DfBQAAQBAJ.

[5] Hoover JD, Whitehead CJ (1975). An experiential-cognitive methodology in the first course in management: some preliminary results. https://absel-ojs-ttu.tdl.org/absel/article/view/2787.

[6] Dewey J (1938). Experience and education. https://philpapers.org/rec/DEWEAE-2.

[7] Burch GF, Giambatista R, Batchelor JH, Burch JJ, Hoover JD (2019). A meta‐analysis of the relationship between experiential learning and learning outcomes. *Decision Sci J Innov Edu*.

[8] Schreck CM, Weilbach JT, Reitsma GM (2020). Improving graduate attributes by implementing an experiential learning teaching approach: a case study in recreation education. J Hosp Leis Sport Tour Educ.

[9] Tomkins L, Ulus E (2016). ‘Oh, was that “experiential learning”?!’ Spaces, synergies and surprises with Kolb’s learning cycle. Manag Learn.

[10] Hajshirmohammadi A (2017). Incorporating experiential learning in engineering courses. IEEE Commun Mag.

[11] Coker JS, Heiser E, Taylor L, Book C (2017). Impacts of experiential learning depth and breadth on student outcomes. J Exp Educ.

[12] Sokhanvar Z, Salehi K, Sokhanvar F (2021). Advantages of authentic assessment for improving the learning experience and employability skills of higher education students: a systematic literature review. Stud Educ Eval.

[13] Kreber C, Klampfleitner M, McCune V, Bayne S, Knottenbelt M (2007). What do you mean by “authentic”? A comparative review of the literature on conceptions of authenticity in teaching. Adult Educ Q.

[14] Palmer P (1998). Exploring the Inner Landscape of a Teacher’s Life.

[15] Lebow DG, Wager WW (1994). Authentic activity as a model for appropriate learning activity: implications for emerging instructional technologies. Can J Learn Technol.

[16] Brown AL, Campione JC (2020). Classroom Lessons.

[17] Herrington A, Herrington J (2005). What is an authentic learning environment? authentic learning environments in higher education.

[18] Sternberg RJ, Wagner RK, Okagaki L (2018). Mechanisms of Everyday Cognition.

[19] Delacre M, Lakens D, Leys C (2022). Correction: why psychologists should by default use Welch’s t-test instead of Student’s t-test. Int Rev Soc Psychol.

[20] Ruxton GD (2006). The unequal variance t-test is an underused alternative to Student’s t-test and the Mann–Whitney U test. Behav Ecol.

[21] Gillett-Swan J, Baroutsis A (2024). Student voice and teacher voice in educational research: a systematic review of 25 years of literature from 1995–2020. Oxf Rev Educ.

[22] Kost RG, de Rosa JC (2018). Impact of survey length and compensation on validity, reliability, and sample characteristics for ultrashort-, short-, and long-research participant perception surveys. J Clin Transl Sci.

[23] Hofkens TL, Ruzek E (2019).

[24] Deslauriers L, McCarty LS, Miller K, Callaghan K, Kestin G (2019). Measuring actual learning versus feeling of learning in response to being actively engaged in the classroom. Proc Natl Acad Sci USA.

[25] Maya J, Luesia JF, Pérez-Padilla J (2021). The relationship between learning styles and academic performance: consistency among multiple assessment methods in psychology and education students. Sustainability.

[26] Wu MJ, Zhao K, Fils-Aime F (2022). Response rates of online surveys in published research: a meta-analysis. Comput Hum Behav Rep.

[27] Leslie LL (1972). Are high response rates essential to valid surveys?. Soc Sci Res.

[28] Nulty DD (2008). The adequacy of response rates to online and paper surveys: what can be done?. Assess Eval High Educ.

[29] Parkin H, Heron E (2023). Listening works: using the listening rooms methodology to explore diversity. JLDHE.

[30] Schimmer T (2014). The case for confidence. ASCD. https://ascd.org/el/articles/the-case-for-confidence?form=MG0AV3.

[31] RMoss K (2004). Confidence: How Winning Streaks and Losing Streaks Begin and End.

[32] Cooper KM, Downing VR, Brownell SE (2018). The influence of active learning practices on student anxiety in large-enrollment college science classrooms. *Int J Stem Educ*.

[33] Imawan OR, Ismail R (2022). Student’s self-confidence change through the application of the guided discovery learning model.

[34] Kew SN, Tasir Z, Kew SN, Tasir Z (2022). Learning analytics in online learning environment: a systematic review on the focuses and the types of student-related analytics data. Tech Know Learn.

[35] Archer M, Morley DA, Souppez J (2020). Real world learning and authentic assessment. Applied pedagogies for higher education: Real world learning and innovation across the curriculum.

[36] Ajjawi R, Tai J, Dollinger M, Dawson P, Boud D (2024). From authentic assessment to authenticity in assessment: broadening perspectives. Assess Eval High Educ.

