# Supplementary material for: Effect of laboratory manual layout: does experiential learning benefit from authentic context?
Source: Access Microbiol. 2025 Jun 17;7(6):000955.v4. doi: 10.1099/acmi.0.000955.v4 (PMC12174585; doi:10.1099/acmi.0.000955.v4)
Supplement: Uncited Supplementary Material 1. [file acmi-7-00955-s001.pdf]

Week two/three: Experiment 1.1

EXPERIMENT 1.1: SPECTROPHOTOMETRIC DETERMINATION OF CYTOCHROME C: PREPARING A CALIBRATION GRAPH

**Aim of the experiment**	To determine the concentration of a coloured compound in a solution by comparison of its light absorption with the light absorption of a standard solution of known concentration.

**Your learning objectives**	1. To learn how to use a simple spectrophotometer to determine the degree of light absorption by a coloured compound.<br>2. To plot a spectrum of light absorbance against wavelength and to determine the wavelength where absorbance is greatest.<br>3. To make up a set of standard solutions of known concentration and to use them to plot a graph of light absorbance against concentration (calibration graph).<br>4. To understand the use of a calibration graph.<br>5. To measure the concentration of an unknown using this graph.<br>6. To consider what degree of precision can be expected from this method of determination of concentration.<br>7. To practise writing up an experiment in your lab book

INTRODUCTION

This experiment uses spectroscopy to measure the concentration of cytochrome c (cyt c). This is a small protein which has an organic molecule, haem, attached to it. It is found in nearly all cells (animal, plant and bacteria) where it acts as an electron carrier in metabolism. There are a number of haem-protein complexes in nature and all of them absorb light in the 380-450 nm region.

Remember: BIOL1302 and MICR1320 students will complete this class in week two, BIOL1303 students will complete it in week three. Please check the class schedule on Minerva, and your personal timetable so you know which session to attend

PREPARATION

- Before you come to the lab, you should read the background materials on Minerva, read through the experimental protocol, and using the information from these sources, complete the preparation MRQ questions. The MRQs will **only** be available **before** the practical session, and marks for these MRQs contribute towards your final module mark.
- Remember that there are resources on Minerva to help you with dilution calculations – use these *before* the practical, so that you are confident in calculating the concentration of cytochrome c in your samples.
- You should also complete other appropriate preparation tasks (see Preparing for Practicals, and the practical checklist), so that you are ready to start your experiment as soon as you arrive.
- You will find it helpful to write a 100-150 word summary of the material and bring it to the lab with you to remind you of the key points from the background material – you can stick this in your lab book between the Title and Aim.

Note – the aim provided for each experiment is written in very general terms. Make sure that your aim is specific to the experiment you are performing (e.g. for this experiment, which coloured compound are you studying?)

11

Week two/three: Experiment 1.1

EXPERIMENT

You are provided with a stock solution of cytochrome c (cyt c) in water containing 0.1 mg/ml. Use this to determine the concentration of cytochrome c in the unknown solution X.

Complete your lab book, including answers to the questions in the Discussion section before you leave. *Hand your book in to your demonstrator.*

METHOD

1. **Determination of peak wavelength: plotting absorption spectrum**

- Collect 2 cuvettes from the end of the bench.
- Fill one cuvette with approximately 3 mL of deionised water to act as a reference, and another with a similar volume of **the stock solution of cyt c (0.1 mg/mL)**. *Instructions for use of the spectrophotometer will be found near the instrument. Be sure to keep both the cuvettes clean on the outside, and to hold them only near the top, away from the region through which the light beam passes.*
- Set the spectrophotometer to 380 nm, zero it with the reference (water) cuvette, and then measure the absorbance of the cyt c solution.
- Now change the wavelength to 385 nm, zero again with the reference tube, and measure the absorbance of the cyt c solution.
- In this way, measure the absorbance at 5 nm intervals, from about 380 nm to 420 nm. *It is imperative that you zero the instrument with the water reference at each wavelength. This is because the light source in the spectrophotometer emits different amounts of light at different wavelengths.*

It is important to use the same instrument for the rest of the experiment, as wavelength settings may vary slightly from instrument to instrument.

RESULTS

Record your results directly into a suitable table in your lab book. Plot the absorption spectrum that you have measured, draw a smooth curve through the points, and hence find the wavelength of maximum absorbance. **Note your observations**; how many peaks are observed in the wavelength range investigated? Are peak(s) symmetrical? *Take additional readings if necessary (if the peak is not symmetrical) to determine the peak wavelength precisely.*

2. **Calibration graph**

The absorbance of a substance in solution is directly dependent on its concentration (Beer-Lambert Law). If the molar absorption coefficient is known (i.e. the absorbance/cm of a 1 M solution at a given wavelength), it should be possible to determine the concentration of an unknown solution, simply by measuring its absorbance. However, this approach is not always accurate because of limitations with the spectrophotometer and the fact that some compounds deviate slightly from the Beer-Lambert Law, particularly at higher concentrations.

A safer way to determine the concentration of an unknown solution is to compare the absorbance of the unknown with that of a series of known concentrations **under the same conditions**. This series of readings is used to produce a **calibration graph** (absorbance v. concentration). This may also be referred to as a standard graph/curve.

Included in any such series is a **blank** or **background** sample, which consists of the solvent (and any reagent used) without any of the substance you are measuring. Any absorbance given by this sample must be subtracted from all the other readings. The lines will then go through zero: no substance – no absorbance.

12

Supplementary Figure 1 – Example excerpt from the NI practical manual. This manual contains a traditional week-by-week format of theoretical and practical information. Please note the page numbers are sequential.

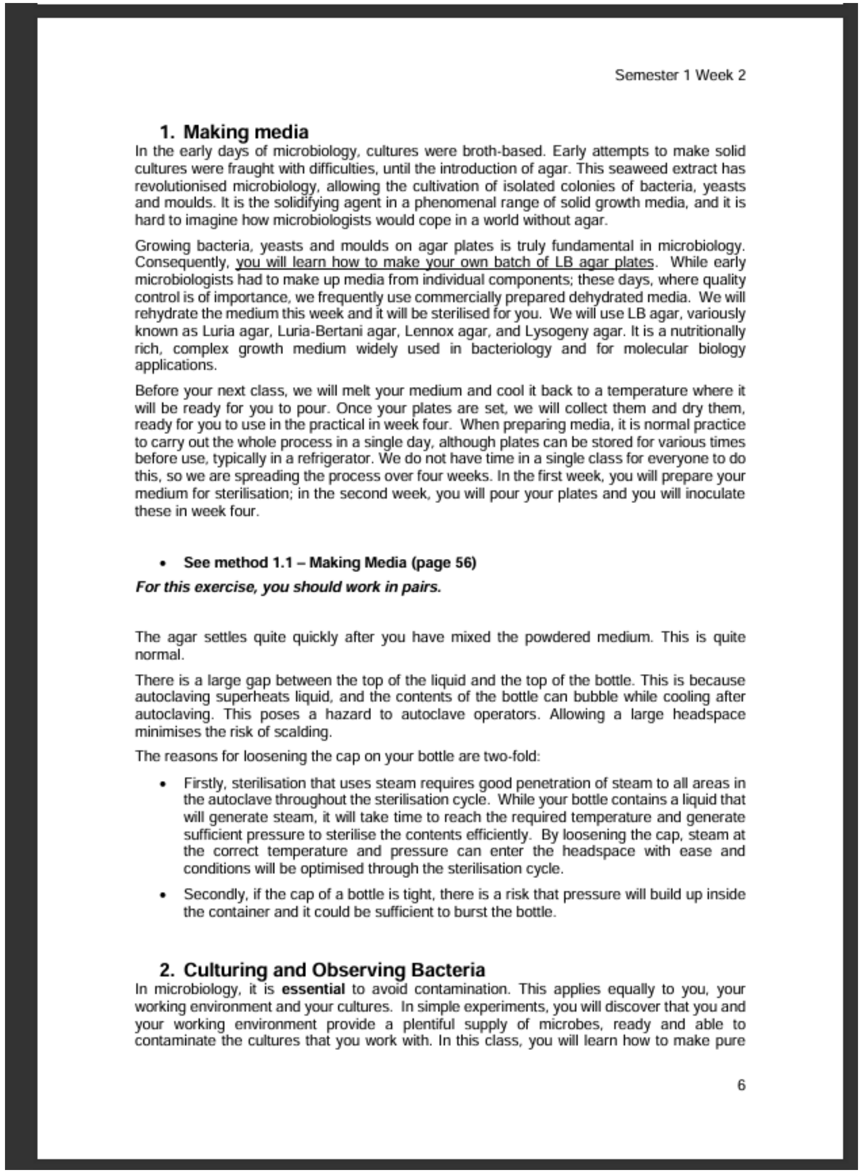
Semester 1 Week 2

1. Making media

In the early days of microbiology, cultures were broth-based. Early attempts to make solid cultures were fraught with difficulties, until the introduction of agar. This seaweed extract has revolutionised microbiology, allowing the cultivation of isolated colonies of bacteria, yeasts and moulds. It is the solidifying agent in a phenomenal range of solid growth media, and it is hard to imagine how microbiologists would cope in a world without agar.

Growing bacteria, yeasts and moulds on agar plates is truly fundamental in microbiology. Consequently, you will learn how to make your own batch of LB agar plates. While early microbiologists had to make up media from individual components; these days, where quality control is of importance, we frequently use commercially prepared dehydrated media. We will rehydrate the medium this week and it will be sterilised for you. We will use LB agar, variously known as Luria agar, Luria-Bertani agar, Lennox agar, and Lysogeny agar. It is a nutritionally rich, complex growth medium widely used in bacteriology and for molecular biology applications.

Before your next class, we will melt your medium and cool it back to a temperature where it will be ready for you to pour. Once your plates are set, we will collect them and dry them, ready for you to use in the practical in week four. When preparing media, it is normal practice to carry out the whole process in a single day, although plates can be stored for various times before use, typically in a refrigerator. We do not have time in a single class for everyone to do this, so we are spreading the process over four weeks. In the first week, you will prepare your medium for sterilisation; in the second week, you will pour your plates and you will inoculate these in week four.

- **See method 1.1 – Making Media (page 56)**

For this exercise, you should work in pairs.

The agar settles quite quickly after you have mixed the powdered medium. This is quite normal.

There is a large gap between the top of the liquid and the top of the bottle. This is because autoclaving superheats liquid, and the contents of the bottle can bubble while cooling after autoclaving. This poses a hazard to autoclave operators. Allowing a large headspace minimises the risk of scalding.

The reasons for loosening the cap on your bottle are two-fold:

- Firstly, sterilisation that uses steam requires good penetration of steam to all areas in the autoclave throughout the sterilisation cycle. While your bottle contains a liquid that will generate steam, it will take time to reach the required temperature and generate sufficient pressure to sterilise the contents efficiently. By loosening the cap, steam at the correct temperature and pressure can enter the headspace with ease and conditions will be optimised through the sterilisation cycle.
- Secondly, if the cap of a bottle is tight, there is a risk that pressure will build up inside the container and it could be sufficient to burst the bottle.

2. Culturing and Observing Bacteria

In microbiology, it is **essential** to avoid contamination. This applies equally to you, your working environment and your cultures. In simple experiments, you will discover that you and your working environment provide a plentiful supply of microbes, ready and able to contaminate the cultures that you work with. In this class, you will learn how to make pure

6

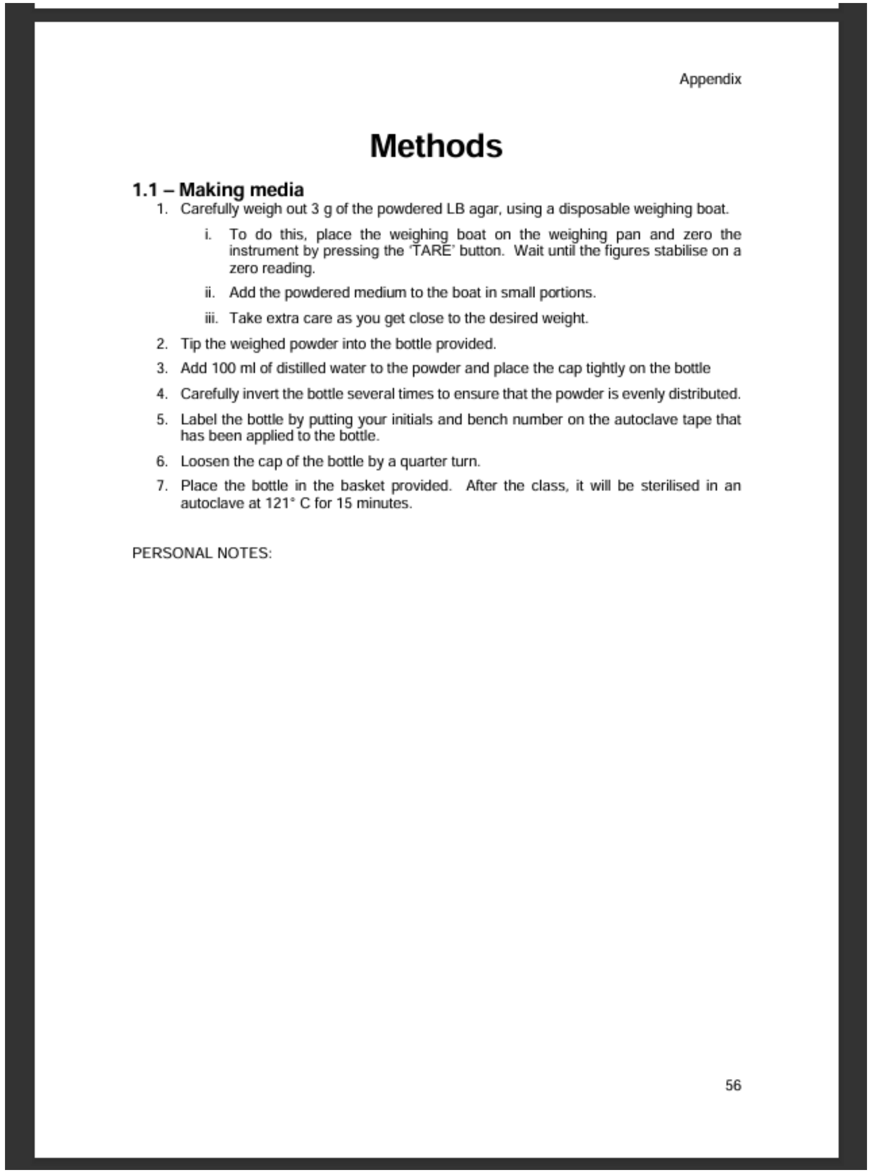
Appendix

Methods

1.1 – Making media

1. Carefully weigh out 3 g of the powdered LB agar, using a disposable weighing boat.
   i. To do this, place the weighing boat on the weighing pan and zero the instrument by pressing the 'TARE' button. Wait until the figures stabilise on a zero reading.
   ii. Add the powdered medium to the boat in small portions.
   iii. Take extra care as you get close to the desired weight.
2. Tip the weighed powder into the bottle provided.
3. Add 100 ml of distilled water to the powder and place the cap tightly on the bottle
4. Carefully invert the bottle several times to ensure that the powder is evenly distributed.
5. Label the bottle by putting your initials and bench number on the autoclave tape that has been applied to the bottle.
6. Loosen the cap of the bottle by a quarter turn.
7. Place the bottle in the basket provided. After the class, it will be sterilised in an autoclave at 121° C for 15 minutes.

PERSONAL NOTES:

56

Supplementary Figure 2 – Example excerpt from the I practical manual. This manual contains a separate methods section in the appendix which contained all experimental information. Each method had an ID number and space for personal notes. Please note the page numbers are not sequential.

A.

Year	Sample size (h)	Lowest value (h)	Lower quartile (Q1) (h)	Mean (h)	Median (h)	Upper quartile (Q3) (h)	Highest value (h)
**23/24**	96	11.4	32.7	56.8 *	54.2	71.8	249.7
**22/23**	104	11.5	32.3	47.5 *	44.1	60.8	117.9

B.

	Sample size	Lowest value %	Lower quartile (Q1) %	Mean %	Median %	Upper quartile (Q3) %	Highest value %
**Intervention**	91	20	50	59.7 $^{n.s}$	55	75	100
**No Intervention**	91	25	47.5	64.8 $^{n.s}$	65	80	100

Supplementary Data Table 1 - Data points represented in manuscript figures 1 and 2. Table A, student engagement with the online learning environment. Table B, student outcomes of practical skills assessment. * P Value <0.05, $^{n.s}$ P Value >0.05
